# Is the disease risk and penetrance in Leber hereditary optic neuropathy actually low?

**DOI:** 10.1016/j.ajhg.2022.11.014

**Published:** 2022-12-23

**Authors:** David A. Mackey, Jue-Sheng Ong, Stuart MacGregor, David C. Whiteman, Jamie E. Craig, M. Isabel G. Lopez Sanchez, Lisa S. Kearns, Sandra E. Staffieri, Linda Clarke, Myra B. McGuinness, Wafaa Meteoukki, Sona Samuel, Jonathan B. Ruddle, Celia Chen, Clare L. Fraser, John Harrison, Neil Howell, Alex W. Hewitt

**Affiliations:** 1 Menzies Institute for Medical Research, School of Medicine, University of Tasmania, Hobart, 7000 TAS, Australia; 2 The University of Western Australia, Centre for Ophthalmology and Visual Science, Lions Eye Institute, Nedlands, 6009 WA, Australia; 3 Centre for Eye Research Australia, Royal Victorian Eye and Ear Hospital, East Melbourne, 3002 VIC, Australia; 4 Statistical Genetics Laboratory, Population Health Department, QIMR Berghofer Medical Research Institute, Brisbane, 4006 QLD, Australia; 5 Cancer Control Group, QIMR Berghofer Medical Research Institute, Brisbane, 4006 QLD, Australia; 6 Flinders Medical Centre, Flinders University, Bedford Park, SA 5042, Australia; 7 Ophthalmology, University of Melbourne, Department of Surgery, Parkville, 3010 VIC, Australia; 8 Save Sight Institute, Discipline of Ophthalmology, Faculty of Health and Medicine, The University of Sydney, Sydney, 2000 NSW, Australia; 9 Department of Ophthalmology, Royal Brisbane and Women's Hospital, Herston, 4006 QLD Australia; 10 Neil Howell, San Diego, CA, USA

**Keywords:** LHON, mitochondria, mtDNA, prevalence, UK Biobank, myocilin, haplogroup, m.11778G>A, m.14484T>C

## Abstract

Pedigree analysis showed that a large proportion of Leber hereditary optic neuropathy (LHON) family members who carry a mitochondrial risk variant never lose vision. Mitochondrial haplotype appears to be a major factor influencing the risk of vision loss from LHON. Mitochondrial variants, including m.14484T>C and m.11778G>A, have been added to gene arrays, and thus many patients and research participants are tested for LHON mutations. Analysis of the UK Biobank and Australian cohort studies found more than 1 in 1,000 people in the general population carry either the m.14484T>C or the m.11778G>A LHON variant. None of the subset of carriers examined had visual acuity at 20/200 or worse, suggesting a very low penetrance of LHON. Haplogroup analysis of m.14484T>C carriers showed a high rate of haplogroup U subclades, previously shown to have low penetrance in pedigrees. Penetrance calculations of the general population are lower than pedigree calculations, most likely because of modifier genetic factors. This Matters Arising Response paper addresses the Watson et al. (2022) Matters Arising paper, published concurrently in *The American Journal of Human Genetics*.

## Main text

We thank Watson and colleagues for a valuable extension of our work on establishing risk of vision loss from Leber hereditary optic neuropathy (LHON).^1^ They show that the risk of vision loss for LHON mutation carriers in the whole population is possibly five times lower than the low rate we found in our extended LHON pedigree study.^2^ They have identified a general population rate of carrying an LHON mutation of 1 in 801, which is more than five times greater than the rate of 1 in 4,600 we estimated from pedigree data.

Watson and colleagues’ low risk of vision loss is consistent with our pedigree study findings where certain mtDNA haplotypes convey a lower risk, as does distance from the nearest affected relatives. This is key for genetic counseling where increasing genomic analysis for other diseases will mean many people will be identified with coincidental LHON mtDNA mutations. While Watson and colleagues found the overall (male and female) penetrance to be 1.11%, it is important to appreciate this is the predicted penetrance from all cases—most of which are familial—and the risk for a person with an incidental finding and no known affected matrilineal relatives is probably even lower.

### Pathogenic LHON mtDNA mutations in other populations

To further explore this issue, we investigated prevalence of LHON-associated variants in two other population cohorts: the UK Biobank (UKB)^3^ and the QSkin study in Australia.^4^ The UKB contains genotypes of 488,377 participants from the UK, while the QSkin study contains genetic data from 43,794 Australian individuals. All study participants provided written informed consent. The UK Biobank was approved by the National Research Ethics Service Committee North West and the QSkin study was approved by the Human Research Ethics Committee at the QIMR Berghofer Medical Research Institute. mtDNA genotypes from the UKB were analyzed with the UK Biobank axiom array. Post-quality-control (QC) analysis of mtDNA data consisted of both nominated disease-associated and common population variants available for 265 markers.^5^ We first constrained our analysis to limit population stratification by removing individuals of non-White British ancestry on the basis of nuclear genetic principal components, resulting in 406,732 participants for this analysis. We then identified participants carrying the following mtDNA variants: m.3460G>A, m.11778G>A, and m.14484T>C (Table 1). In the UKB dataset, we did not observe any carriers of the m.3460G>A mutation.

**Table 1:** Rate of mtDNA mutation carriers in UKB (number of minor allele carriers from N = 406,732), QSkin2 (number of minor allele carriers from N = 8,029), and Watson paper (number of minor allele carriers from N = 4,012)

**LHON mutation**	**UKB cases (% m)**	**UKB% in pop**	**UKB frequency**	**QSkin2 cases (% m)**	**QSkin2% in pop**	**QSkin2 frequency**	**Watson cases**	**Watson% in pop**	**Watson frequency**
m.3460G>A	nil	0%	N/A	nil	0%	N/A	0	0%	N/A
m.11778G>A	137 (39% m)	0.034%	1 in 2,969	3 (0% m)	0.037%	1 in 2,676	1	0.025%	1 in 4,012
m.14484T>C	392 (40% m)	0.096%	1 in 1,038	7 (57% m)	0.0875%	1 in 1,143	4	0.0997%	1 in 1,003
All mutations	529 (40% m)	0.130%	1 in 769	10 (40% m)	0.125%	1 in 803	5	0.125%	1 in 802

Pop, population; m, male; f, female.

mtDNA genotypes were available from a large Australian study, QSkin, which was designed to identify genetic risk factors for skin cancer. The later tranche of genetic analysis, “QSkin2,” used the GSA chip that included both the m.14484T>C and m.11778G>A variants. Of 8,029 people analyzed with the GSA chip, there were three (all female) carriers of the m.11778G>A variant and seven (four male, three female) carriers of the m.14484T>C variant. This gives an approximate carrier frequency of 3 in 8,029 or 0.037% (1 in 2,676) for m.11778G>A and 7 in 8,029 or 0.087% (1 in 1,143) for m.14484T>C (Table 1). Overall, we found a very similar population carrier rate for the two mutations in the three studies (Table 1). All studies suggest more than 1 in 1,000 people carry one of the two main pathogenic LHON mutations.

### mtDNA haplogroup frequencies associated with LHON mutations

Yonova-Doing and colleagues^5^ noted that in the UKB, the m.11778 G>A variant occurred proportionately across all nine main European haplogroups. However, the odds of carrying m.14484 T>C on the J macro-haplogroup was lower (OR [95% CI], 0.31 [0.18–0.55], p = 6.4 × 10^−5^), whereas on the U macro-haplogroup, the odds were higher (OR [95% CI], 1.58 [1.38–1.80], p = 1.8 × 10^−11^). This was surprising as all three cases of m.14484 T>C in a large study of cord blood in the UK^6^ were on haplogroup J, and at least 34% of Australian men with vision loss due to LHON from any mutation are on haplogroup J.^2^ These findings add to the growing evidence that haplogroup J influences the clinical penetrance of LHON.^7^ The observation that fewer m.14484T>C carriers are haplotype J than the proportion of J haplotypes in the general population suggests that there may be a large non-J founder effect in the UK. This would account for a significant proportion of m.14484T>C carriers. Most likely this is a U haplogroup founder effect (see Figures S10F and S10G from Yonova-Doing^5^).

**Figure 1:**
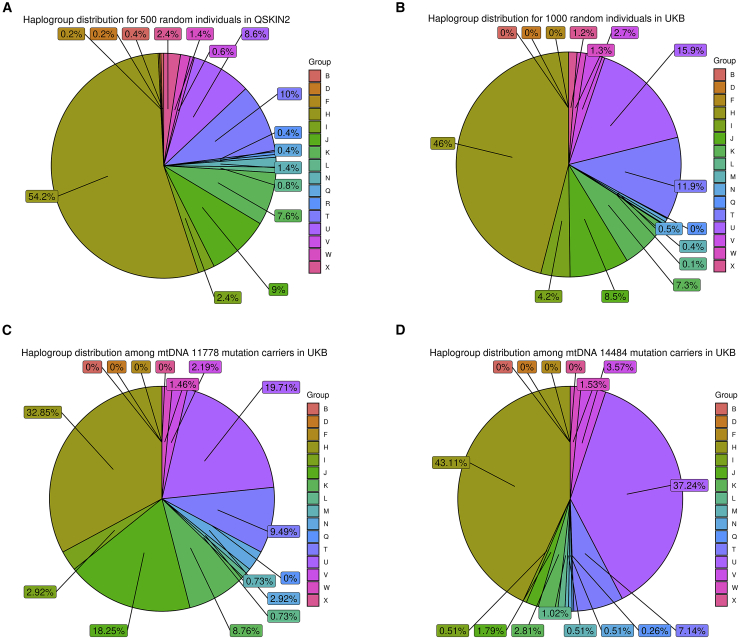
Pie charts of main haplogroups frequencies in QSkin2 (500), UKB (1,000), UKB m.11778G>A, and UKB m.14484T>C (A) Pie chart of haplotype distribution for 500 random individuals in QSkin2. (B) Pie chart of haplotype distribution for 1,000 random individuals in UKB. (C) Pie chart of haplotype distribution among m.11778G>A carriers in UKB. (D) Pie chart of haplotype distribution among m.14484T>C carriers in UKB.

In addition, we analyzed the mitochondrial haplogroups by using the haplogrep online interactive platform (https://haplogrep.i-med.ac.at/app) in 500 individuals selected randomly from the QSkin2 database and 1,000 random individuals from the UKB and the m.11778G>A and m.14484T>C carriers from the UKB and QSkin2 studies (Figure 1). As previously identified,^5^ there is a disproportionate number of individuals belonging to haplogroup U among the UKB m.14484T>C carriers. Although the percentage of haplogroup U in QSkin2 (8.6%) was less than UKB (15.9%), for the seven identified m.14484T>C carriers in QSkin2, three (43%) were haplotype U compared to 37% (145 individuals) of m.14484T>C carriers in UKB.

### Further analysis of haplogroup U

For the QSkin2 m.14484T>C carriers, the most common haplogroup was U:3/7 belong to U5a1a1. This haplogroup was found in 19/500 (4%) random individuals in the QSKIN2 and 64/1,000 (6%) random UKB controls. A full breakdown of haplogroup U in the UKB is given in Table 2. The most frequent subclades associated with m.14484T>C carriers were the following: U5ab (n = 57), U5a1a1 (n = 36), U5b2 (n = 29), U5a (n = 6), and U5a1+@16,192 (n = 6). Of particular note is the presence of U5a1a1. This subclade was found in three Australian pedigrees: VIC29 with the m.11778G>A and a low penetrance documented at 3%; NSW15 with the m.14484T>C and a low penetrance documented at 8%; and VIC20, which not only had the m.14484T>C but also the m.11778G>A variant yet penetrance of 7%.

**Table 2:** Subclade assignment within haplogroup U for the UKB genotypes

**Assigned subclade (within U′)**	**Number of UKB individuals in the subclade**			
	**Random 1,000 individuals set**	**m.14484T>C carriers**	**m.11778G>A carriers**	**Participants with eye examination**
U	1	–	–	–
U1a	1	–	–	–
U2′3'4′7′8′9	2	–	–	–
U2e	9	3	3	–
U3	11	–	–	1
U4	16	–	2	3
U5a	0	6	–	1
U5a1+@16192	15	6	–	–
U5a1a1	10	36	3	7
U5a1a1+16362	7	1	–	–
U5a1b	11	–	1	3
U5a1d	1	–	–	–
U5a2b4	2	1	–	–
U5a2b4a	1	–	–	–
U5a2c4	1	–	–	1
U5a′b	16	57	1	7
U5b1+16189+@16192	16	2	5	–
U5b1g	0	–	–	–
U5b2	33	29	12	11
U5b2a1a1a	1	–	–	–
U5b3	1	–	–	–
U5b3g^∗^	1	–	–	–
U7	0	1	–	–
U8a	3	–	–	–

Other Australian pedigrees with haplogroup U included the following: QLD01 with the double mutation m.14484T>C and m.4160T>C, with a very high penetrance of 73% and belonging to subclade U4a1a; NSW03 with m.11778G>A, with 8% penetrance and belonging to subclade U5a1; NZ01 with m.11778G>A with one female case belonging to U; NZ02 with m.11778G>A with 19% penetrance and belonging to subclade U5b2a; QLD12 with m.11778G>A with 30% penetrance belonging to U; VIC03 with m.11778G>A with 16% penetrance and belonging to U; and VIC18 with m.11778G>A with 33% penetrance belonging to subclade U4c1.

### Predicted LHON penetrance in the UK and the UKB

Given the reported prevalence of vision loss from LHON in the UK is 1 in 31,000,^8^ and a carrier rate of approximately 1 in 769 people, we calculate an overall penetrance of 2.5%. This is again very similar to the Australian finding by Watson et al.^2^ Although individuals with vision loss from LHON may not have participated in the UKB,^9^ on the basis of the predicted prevalence of LHON vision loss of 1 in 31,000 people in the UK,^8^ we would expect there to have been 13 LHON cases with vision loss.

Visual acuity and fundus photographs were available from 67,040 individuals (16.5%) in the UKB and, of those, two individuals would be expected to have vision loss from LHON. However, obtaining a good quality photograph of a person with reduced vision from LHON is difficult. None of the m.11778G>A or m.14484T>C mutation carriers in the UKB had been coded as having optic atrophy from LHON. Thus, to further investigate affected status in LHON mutation carriers, we assessed visual acuity and corresponding optic disc photographs.

**Figure 2:**
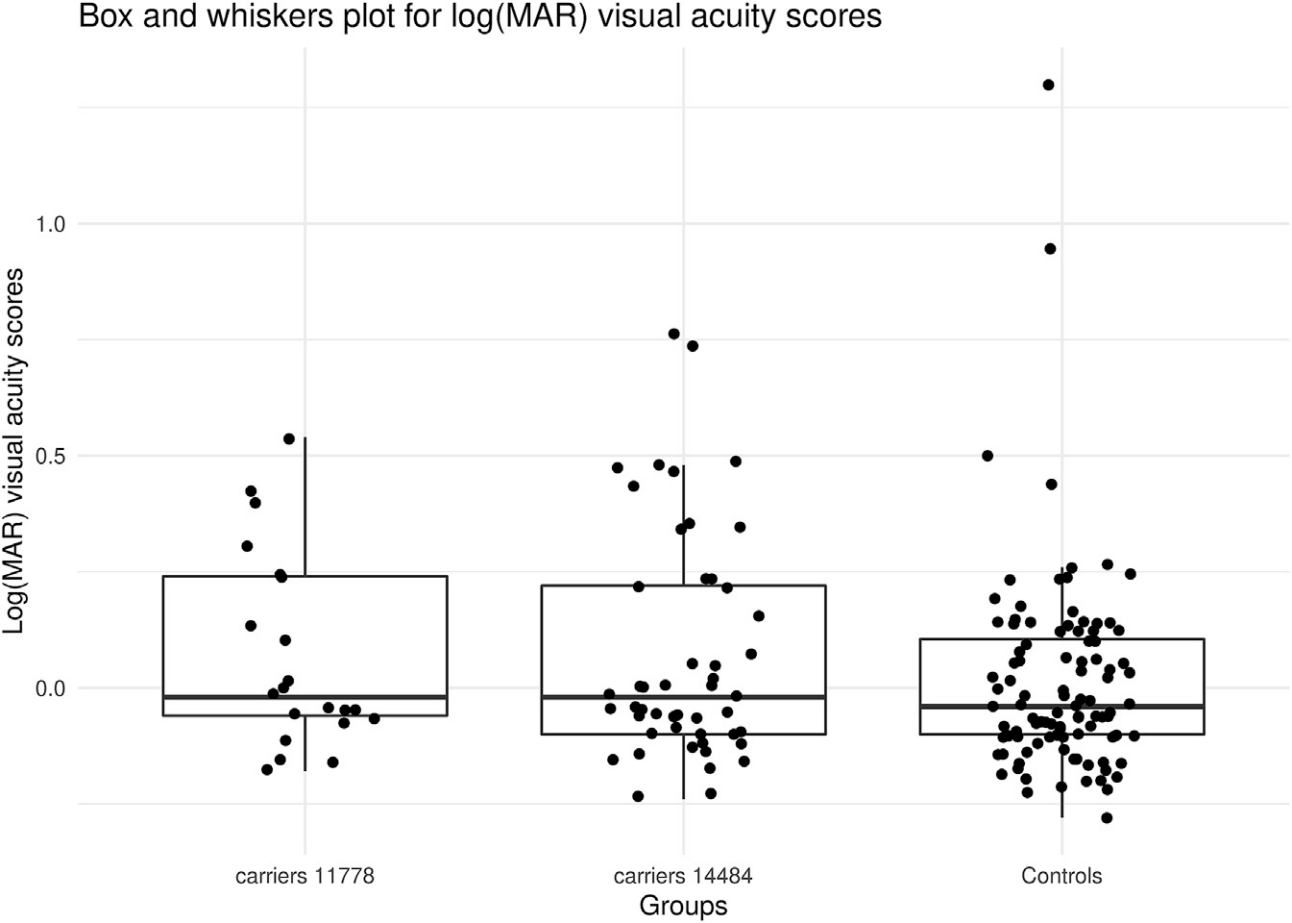
Distribution of visual acuity in individuals who had retinal photographs (controls, m.11778G>A carriers, and m.14484T>C carriers) Box and whisker plots show logMAR visual acuity scores. LogMAR 1.0 = 20/200 and LogMAR 0.0 = 20/20.

Visual acuity was measured in LogMAR and the distribution of visual acuity in those measured is shown in Figure 2. In UKB, 67,040 participants (UKB Field: 21015) had non-stereo fundus images obtained with a Topcon 3D OCT-1000 MKII camera (Topcon Corporation). These images have previously been used to grade cup-to-disc ratio,^10^ however, the ability to grade optic atrophy is very dependent on the flash illumination and pupil dilatation among other factors. Photos were available on 22/137 (16.0%) of the m.11778G>A carriers and 49/392 (12.5%) of the m.14484T>C carriers. None of these carriers had ICD10 coding for optic atrophy (ICD10 H47.2). In addition to the 71 people with either the m.11778G>A or m.14484T>C mtDNA mutations, we included 100 randomly selected healthy controls and three additional non-LHON-mutation carriers from the UKB who had been coded with optic atrophy (ICD10 H47.2). All images were graded in a masked fashion by two independent clinicians who did not know the percentage of individuals with LHON mutations or an optic atrophy diagnosis.

None of the m.11778G>A or m.14484T>C carriers in the UKB had a visual acuity less than 20/200 (LogMAR 1.0). Natural history studies of LHON suggest 50% of people affected by LHON have vision worse than 20/200 (LogMAR 1.0).^11^^,^^12^ As visual acuity in LHON rarely recovers to 20/40 (LogMAR 0.3), we also took this low threshold of visual acuity for our analysis (Table 3). Further clinical details are given in additional clinical information. No m.14484T>C-carrying participant had vision less than 20/200 (LogMAR 1.0) in either eye. There was one possible male case with bilateral reduced vision and optic atrophy noted in one eye and three people with reduced vision in just one eye and optic atrophy noted in the worse eye by one observer. If we consider these four individuals as potentially affected, this would give 4/49 or 8% penetrance for m.14484T>C, although this would be 0% if we used a stricter definition.

**Table 3:** Visual acuities in mtDNA mutation carriers who had retinal photographs

	**Bilateral worse than 6/12 (LogMAR 0.3)**	**Unilateral worse than 6/12 (LogMAR 0.3)**	**Normal 6/12 (LogMAR 0.3) or better**
Controls	0	9 (1 m unilateral OA)	91 (6 unilateral OA 1 m 5 f and 1 m bilateral OA, both observers)
m.11778G>A	1^a^ (1 m bilateral OA)	5 (1 m unilateral OA)	16 (4 unilateral OA 3 m 1 f, both observers)
m.14484 T>C	4 (1 m unilateral OA)	9 (3 unilateral OA 1 m 2 f)	36 (1 f unilateral, OA both observers)
Optic Atrophy	0	1 (1 m bilateral OA)	2 (1 m bilateral OA, and 1 m unilateral, OA both observers)

OA, optic atrophy; m, male; f, female.

a Participant did not have vision recorded.

### m.14484T>C is the most common mutation in the general population but a less frequent mutation among reported LHON cases

In Australia,^2^ 20% of LHON vision loss cases were due to m.14484T>C; however, in the UK m.14484T>C only accounted for 25/211 (12%) of the affected men within LHON pedigrees in the UK/Eire^13^ or 3/63 (4.8%) of men or 3/82 (3.7%) of men and women in North East England with vision loss from LHON.^8^ The UKB data suggest that the m.14484T>C mutation is nearly three times more common than the m.11778G>A mutation in the UK population, but it appears to account for only 4.8%–12% of male LHON cases in the UK. We could not find any case of optic atrophy with severely reduced vision in the UKB, implying that the m.14484T>C must have a much lower penetrance than m.11778G>A in the UK. The predominance of m.14484T>C in UKB participants and complete lack of UKB participants with the m.3460G>A matches published pedigree experience with these mutations. With the exception of pedigree “706” in Denmark that traces back to 1842,^14^ there are no very large LHON pedigrees with the m.3460G>A mutations.^13^ In contrast, the m.14484T>C mutation is found in a mega Dutch/Quebecois pedigree that extends back to before the year 1600.^15^ The high percentage of m.14484T>C in the UKB with haplogroups U5a1a1 and U5a′b could also be due, in part, to a founder effect of a very low penetrance LHON pedigree.

### Similar low penetrance findings in other optic nerve disease

Interestingly, this observation of lower penetrance of a disease in the general population compared to pedigree studies has been documented in another optic nerve disease, glaucoma, associated with the p.Gln368Ter (rs74315329) risk allele in the myocilin gene (*MYOC*).

As part of the Glaucoma Inheritance Study Tasmania (GIST), eight pedigrees carrying the *MYOC* p.Gln368Ter risk allele were identified from 1,730 consecutive cases of primary open-angle glaucoma (POAG). We found that age-related penetrance for ocular hypertension or POAG was 72% at 40 years of age and 82% at age 65 years of age.^16^ In parallel, from the Blue Mountains Study cohort of 2,142 people, we identified two people who were unaffected at the time but were carriers of *MYOC* p.Gln368Ter, suggesting a carrier frequency of 1 in 1,071.^17^ Subsequently, cross-sectional studies of the UKB and the Australian and New Zealand Registry of Advanced Glaucoma (ANZRAG) reported *MYOC* p.Gln368Ter in 1 in 393 (0.25%) participants of the UKB (n = 411,337) but 5.77% (174/3,071) of the ANZRAG. The penetrance of the *MYOC* p.Gln368Ter variant was 7.6% in people with glaucoma (based on self-report or International Classification of Diseases) in the UKB, but the penetrance of glaucoma was 56.1% in the ANZRAG.^18^ A subsequent UKB study using optic disc-defined glaucoma found 1 in 4 of the individuals with *MYOC* p.Gln368Ter had evidence of glaucoma.^19^ In addition, combination with a polygenic risk score for glaucoma increased the prevalence of glaucoma.

From the experience with glaucoma genetics and penetrance, we would expect other genetic modifiers, such as the already established mtDNA haplotype and other as yet unidentified genetic variants, would influence LHON penetrance. LHON research should focus on identifying these modifiers, which would expand our understanding of the disease and enable better informed reproductive choices, as well as facilitate the recruitment of individuals at high risk of LHON vision loss into prevention trials, including mitochondrial donation, and thus improve the power of these studies to identify effective treatments.

### Conclusion

Our new analysis of the UKB and QSkin2 cohorts indicates a prevalence of LHON mutations greater than 1 in 1,000 in the general population, as Watson et al. have found. On the basis of the clinical data from the UKB and the UK population data, we agree that the penetrance in the general population appears around 2.5%, much lower than the 17% risk for males that we quoted in our Australian LHON pedigree paper. Clinical examinations of UKB m.11778G>A and m.14484T>C carriers did not identify any cases of severe vision loss (worse than 20/200) consistent with being affected by LHON and only 4/49 m.14484T>C carriers had bilateral vision less than 20/40. Establishing penetrance from any pedigree study will tend to be biased to families carrying other modifiers—whether this be mitochondrial haplotype or polygenic risk score—that increase prevalence. The main explanation for this difference in the case of LHON would appear to be a large founder effect of pedigrees with the m.14484T>C on low-risk haplogroup U subclade backgrounds in the UK and Australian population. Providing genetic counseling to people identified with m.11778G>A or m.14484T>C requires a well-ascertained matrilineal family history for vision loss as well as an analysis of mitochondrial haplogroup/subclades to provide a more accurate risk prediction for vision loss.

### Additional clinical information

Of the controls with reduced vision, no person had bilateral reduced vision. Of the nine eyes (from four male and five female) with reduced vision in the control group, only one eye (male) was graded as optic atrophy by one observer. The photographs in the UKB are not all of high quality; in the control group, both eyes of six participants and one eye of 15 participants were reported as ungradable by both observers. Of those controls with normal vision, one was classified by both observers as having bilateral optic atrophy and six were classified by both observers as having unilateral optic atrophy.

Three male cases of optic atrophy based on ICD10 H47.2 (none of whom had an LHON mutation) were classified as having bilateral optic atrophy and one having as unilateral optic atrophy; however, only one eye was worse than 6/12: LogMAR (R 0.18, L 0.08), LogMAR (R 0.26, L 0.34), LogMAR (R 0.04, L 0.3).

Of the 22 participants with the m.11778G>A mutation, there were two potentially affected male participants. One male participant had no recorded visual acuity, possibly because they were unable to read the top line of the chart at the testing distance, but optic atrophy in both eyes was noted by both observers. This person had no other ICD10 codes recorded. One male participant had reduced vision LogMAR (R 0.4, L 0.18) and optic atrophy in the worse eye noted by both observers. The remaining participants with reduced vision were as follows: male LogMAR (R 0.54, L −0.24) with right optic nerve ungradable and left optic nerve normal; male LogMAR (R 0.3, L 0.04) normal optic nerves in both eyes; male LogMAR (R 0.42, L 0.06) ungradable in both eyes; female (R −0.16, L 1.05) right optic nerve normal and left ungradable. Of the participants with normal vision, four (three males, one female) were categorized by both observers as having unilateral optic atrophy. In summary, we could not identify any definitely affected LHON cases in those carrying m.11778G>A. For m.11778G>A, there were two likely male cases, one with bilateral optic atrophy but no recorded visual acuity and one with reduced vision (R 0.4 and L 0.18) and optic atrophy noted in the worse eye. This would give 2/22 or 9% penetrance for m.11778G>A; this may be 0% if more stringent criteria were used.

Of the 49 participants with m.14484T>C, four participants were potentially affected: three males and one female. Four participants had reduced visual acuity in both eyes. Three of these, male (R 0.34, L 0.54), male (R 0.76, L 0.54), and female (R 0.46, L 0.64), had both discs classified as normal by both observers, while the fourth, male (R 0.74, L 0.38), had one disc classified as optic atrophy by one observer. Nine participants had reduced vision in one eye. Of these, three were classified as optic atrophy but only by one grader: female (R 0.48, L −0.02), female (R −0.12, L 0.52), and male (R 0.48, L −0.06). Of participants with vision 20/40 (LogMar 0.3) or better, only one was graded as having optic atrophy in one eye by both observers: female (R 0.22, L −0.08). Given the slightly reduced vision in the eye with optic atrophy, we would classify them as potentially affected. In summary, we could not identify any definitely affected LHON cases in those carrying m.14484T>C.

## References

[1] Watson E.C., Davis R.L., Sue C.M. (2022). Is the disease risk and penetrance in Leber's hereditary optic neuropathy actually low?. Am. J. Hum. Genet..

[2] Lopez Sanchez M.I.G., Kearns L.S., Staffieri S.E., Clarke L., McGuinness M.B., Meteoukki W., Samuel S., Ruddle J.B., Chen C., Fraser C.L. (2021). Establishing risk of vision loss in Leber hereditary optic neuropathy. Am. J. Hum. Genet..

[3] Bycroft C., Freeman C., Petkova D., Band G., Elliott L.T., Sharp K., Motyer A., Vukcevic D., Delaneau O., O'Connell J. (2018). The UK Biobank resource with deep phenotyping and genomic data. Nature.

[4] Olsen C.M., Green A.C., Neale R.E., Webb P.M., Cicero R.A., Jackman L.M., O'Brien S.M., Perry S.L., Ranieri B.A., Whiteman D.C., QSkin Study (2012). Cohort profile: the QSkin Sun and Health Study. Int. J. Epidemiol..

[5] Yonova-Doing E., Calabrese C., Gomez-Duran A., Schon K., Wei W., Karthikeyan S., Chinnery P.F., Howson J.M.M. (2021). An atlas of mitochondrial DNA genotype-phenotype associations in the UK Biobank. Nat. Genet..

[6] Elliott H.R., Samuels D.C., Eden J.A., Relton C.L., Chinnery P.F. (2008). Pathogenic mitochondrial DNA mutations are common in the general population. Am. J. Hum. Genet..

[7] Hudson G., Carelli V., Spruijt L., Gerards M., Mowbray C., Achilli A., Pyle A., Elson J., Howell N., La Morgia C. (2007). Clinical expression of Leber hereditary optic neuropathy is affected by the mitochondrial DNA-haplogroup background. Am. J. Hum. Genet..

[8] Yu-Wai-Man P., Griffiths P.G., Brown D.T., Howell N., Turnbull D.M., Chinnery P.F. (2003). The epidemiology of Leber hereditary optic neuropathy in the North East of England. Am. J. Hum. Genet..

[9] Cumberland P.M., Foster P.J., Rahi J.S. (2015). Understanding visual impairment in UK Biobank. Ophthalmic Physiol. Opt..

[10] Craig J.E., Han X., Qassim A., Hassall M., Cooke Bailey J.N., Kinzy T.G., Khawaja A.P., An J., Marshall H., Gharahkhani P. (2020). Multitrait analysis of glaucoma identifies new risk loci and enables polygenic prediction of disease susceptibility and progression. Nat. Genet..

[11] Newman N.J., Yu-Wai-Man P., Carelli V., Biousse V., Moster M.L., Vignal-Clermont C., Sergott R.C., Klopstock T., Sadun A.A., Girmens J.F. (2021). Intravitreal Gene Therapy vs. Natural History in Patients With Leber Hereditary Optic Neuropathy Carrying the m.11778G>A ND4 Mutation: Systematic Review and Indirect Comparison. Front. Neurol..

[12] Stephenson K.A.J., McAndrew J., Kenna P.F., Cassidy L. (2022). The Natural History of Leber's Hereditary Optic Neuropathy in an Irish Population and Assessment for Prognostic Biomarkers. Neuro Ophthalmol..

[13] Mackey D.A., Oostra R.J., Rosenberg T., Nikoskelainen E., Bronte-Stewart J., Poulton J., Harding A.E., Govan G., Bolhuis P.A., Norby S. (1996). Primary pathogenic mtDNA mutations in multigeneration pedigrees with Leber hereditary optic neuropathy. Am. J. Hum. Genet..

[14] Rosenberg T., Nørby S., Schwartz M., Saillard J., Magalhães P.J., Leroy D., Kann E.C., Duno M. (2016). Prevalence and Genetics of Leber Hereditary Optic Neuropathy in the Danish Population. Invest. Ophthalmol. Vis. Sci..

[15] Howell N., Oostra R.J., Bolhuis P.A., Spruijt L., Clarke L.A., Mackey D.A., Preston G., Herrnstadt C. (2003). Sequence analysis of the mitochondrial genomes from Dutch pedigrees with Leber hereditary optic neuropathy. Am. J. Hum. Genet..

[16] Craig J.E., Baird P.N., Healey D.L., McNaught A.I., McCartney P.J., Rait J.L., Dickinson J.L., Roe L., Fingert J.H., Stone E.M., Mackey D.A. (2001). Evidence for genetic heterogeneity within eight glaucoma families, with the GLC1A Gln368STOP mutation being an important phenotypic modifier. Ophthalmology.

[17] Baird P.N., Richardson A.J., Craig J.E., Rochtchina E., Mackey D.A., Mitchell P. (2005). The Q368STOP myocilin mutation in a population-based cohort: the Blue Mountains Eye Study. Am. J. Ophthalmol..

[18] Han X., Souzeau E., Ong J.S., An J., Siggs O.M., Burdon K.P., Best S., Goldberg I., Healey P.R., Graham S.L. (2019). Myocilin Gene Gln368Ter Variant Penetrance and Association With Glaucoma in Population-Based and Registry-Based Studies. JAMA Ophthalmol..

[19] Zebardast N., Sekimitsu S., Wang J., Elze T., Gharahkhani P., Cole B.S., Lin M.M., Segrè A.V., Wiggs J.L., International Glaucoma Genetics Consortium (2021). Characteristics of p.Gln368Ter Myocilin Variant and Influence of Polygenic Risk on Glaucoma Penetrance in the UK Biobank. Ophthalmology.

